# Evaluation of Antibody Response Directed against Porcine Reproductive and Respiratory Syndrome Virus Structural Proteins

**DOI:** 10.3390/vaccines8030533

**Published:** 2020-09-16

**Authors:** Hung Q. Luong, Huong T. L. Lai, Hiep L. X. Vu

**Affiliations:** 1 Nebraska Center for Virology and Department of Animal Science, University of Nebraska-Lincoln, Lincoln, NE 68583-0900, USA; lqhungpt@gmail.com; 2 Faculty of Veterinary Medicine, Vietnam National University of Agriculture, Hanoi 10000, Vietnam; ltlhuong@vnua.edu.vn

**Keywords:** swine viruses, PRRSV, humoral immunity, luciferase-immunoprecipitation system, antibody profile

## Abstract

Luciferase-immunoprecipitation system (LIPS), a liquid phase immunoassay, was used to evaluate antibody responses directed against the structural proteins of PRRSV in pigs that were experimentally infected with virulent PRRSV strains. First, the viral N protein was used as a model antigen to validate the assay. The LIPS results were highly comparable to that of the commercial IDEXX PRRS X3 ELISA. Subsequently, the assay was applied to simultaneously measure antibody reactivity against all eight structural proteins of PRRSV. The highest immunoreactivities were detected against GP3, M, and N proteins while the lowest reactivity was detected against ORF5a protein. Comparative analysis of the kinetics of antibody appearance revealed that antibodies specific to N protein appeared earlier than antibodies against GP3. Finally, the assay was applied to measure immunoreactivities of clinical serum samples against N and GP3. The diagnostic sensitivity of the LIPS with N protein was superior to that of the LIPS with GP3. Collectively, the results provide additional information about the host antibody response to PRRSV infection.

## 1. Introduction

Porcine reproductive and respiratory syndrome virus (PRRSV) is currently circulating in most swine producing countries, causing substantial economic losses [1]. The viral genome is a positive sense, single stranded RNA molecule of approximately 15kb in length which contains at least 11 open reading frames (ORFs) [2]. ORF1a and ORF1b comprise approximately 80% of the viral genome and encode for at least 14 non-structural proteins (nsp) that are responsible for replication and transcription of the viral genome [3]. In addition, the non-structural proteins are also involved in modulation of the host immunity [3]. The remaining eight ORFs reside in the 3′ end of the viral genome and encode for viral structural proteins. Specifically, ORF2a, ORF3, and ORF4 encode for three minor glycoproteins GP2, GP3, and GP4, respectively [4,5,6,7]. These three minor glycoproteins form heterodimers that are dispensable for viral particle formation but are required for viral infectivity, due to their interaction with CD163, a key receptor for PRRSV entry [8,9,10]. ORF5 and ORF6 respectively encode for GP5 and membrane (M) protein which form heterodimers that are indispensable for viral particle formation [8,11]. ORF7 encodes for the viral nucleocapsid (N) protein responsible for encapsulating the viral RNA genome [12]. ORF2b is embedded within ORF2a and encodes for the envelope (E) protein, an ion-channel protein involved in uncoating of virus and release of the genome in the cytoplasm [13,14]. ORF5a encodes for a newly discovered protein called ORF5a-protein which is translated from an alternative reading frame of the sub-genomic mRNA5 (sgmRNA5) [15]. The initiation codon of ORF5a is 10 nucleotides upstream of the initiation codon of ORF5 but the later ORF is preferentially expressed [15]. ORF5a-protein is required for viral infectivity, but its biological functions remain to be determined [16].

Pigs infected with virulent PRRSV strains develop a robust antibody response that can be detected at 5 days post-infection (dpi) [17]. By 14 dpi, all pigs exposed to PRRSV have seroconverted and antibodies can be detected for up to 300 dpi [17,18]. The intensity of host immune response positively correlates with the virulence of the PRRSV strains to which the hosts are infected [19]. Antibodies developed early after infection are not capable of neutralizing the virus [20]. It has been reported that these non-neutralizing antibodies might enhance viral infection, a phenomenon known as antibody-dependent enhancement (ADE) of infectivity [21]. Neutralizing antibodies are not detected until approximately 4 weeks post-infection and remain at low titers after appearance [20]. Glycan shielding and decoy-epitopes are the possible mechanisms for the virus to escape antibody neutralization [11,22,23]. Neutralizing epitopes were initially identified in the ectodomain of GP5 [22]. Subsequently, it is reported that antibodies against the GP2, GP3, GP4, and M also possess neutralizing activities [24].

Significant effort has been made to characterize the ontogeny of swine humoral immune response to PRRSV infection. Immunoblotting analysis, using PRRSV-infected cell lysate as the target antigens, revealed that PRRSV-infected pigs developed antibodies against three viral major proteins: N (15 KDa), M (19 KDa), and GP5 (25 KDa) [17,18,25]. Antibodies against N protein were consistently detected from 7 dpi and continued to be detected up to at least 300 dpi [17,18]. On the other hand, antibodies against M and GP5 varied among infected pigs and were not be detected until 14 or 35 dpi [17]. These studies demonstrated that N protein is highly immunogenic and is a good target for serodiagnosis [17]. Most commercial ELISAs used for serodiagnosis of PRRSV are developed based on N protein although the exact composition of the antigen targets for these commercial ELISAs is proprietary information [26]. Since GP5-M heterodimer has been suggested to be critical to viral infection and antibody neutralization, protein-specific antibody ELISAs were developed to study antibody responses to 5′ and 3′ termini of GP5 and M protein as well as the chimeric protein containing GP5 and M ectodomain [27]. The results indicated that antibodies directed against these two proteins were detected between 28 and 42 dpi. Pepscan ELISA was used to identify B cell epitopes located in the nsp2 and all viral structural proteins [28]. It was found that nsp2 contained higher frequency of immunodominant epitopes than structural proteins. Of the structural proteins studied, only GP3 and M protein possess peptides that were recognized by 100% (*n* = 15) infected pigs [28]. Protein-specific antibody ELISAs were also used to characterize antibodies directed against multiple PRRSV non-structural proteins including nsp1, nsp2, nsp4, nsp7, and nsp8 [29]. The highest immunoreactivities were against nsp1, nsp2, and nsp7. Interestingly, the diagnostic performance of nsp7 ELISA was highly compatible to that of the commercial ELISA that is based on N as the target antigen [29].

**Figure 1:**
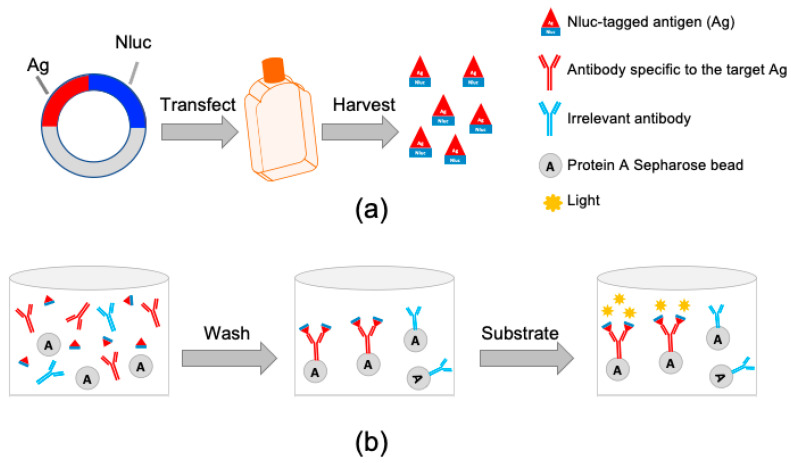
Schematic representation of the luciferase-immunoprecipitation system (LIPS). (**a**) Generation of luciferase-tagged antigens (Ag). Each individual PRRSV structural protein antigen is cloned in-frame to the 5′ terminus of the nanoluc luciferase gene (Nluc). The resulting plasmid is transfected into HEK 293-T cells. At 60 h after transfection, cell lysate containing Nluc-tagged antigen is harvested and used for the LIPS. (**b**) Evaluation of immunoreactivities against the NLuc-tagged antigens. Cell lysate containing Nluc-tagged antigen is incubated with test serum samples together with protein A Sepharose beads in a filter 96-well plate. If the test samples contain antibody (IgG) specific to the Nluc-tagged antigen, the antigen-antibody complexes are formed, which are captured by the protein A Sepharose beads and retained in the well. Unbound NLuc-tagged antigen is washed away. Once the luciferase substrate is added to the well, the Nluc-tagged antigen bound to the bead will react with the substrate and produce luminescence. The light units produced by the Nluc-tagged antigen is proportional to the amount of antigen-specific antibody present in the test serum samples.

While there is a large body of the literature describing the overall humoral immune responses to PRRSV infection, information about antibody response to the viral minor glycoproteins is scarce. Thus, the primary objective of this study was to comparatively evaluate immunogenicity of the PRRSV structural proteins. For this purpose, we adapted a liquid phase immunoassay called the luciferase-immunoprecipitation system (LIPS) to simultaneously measure antibody reactivities against the eight structural proteins in the same serum samples. The LIPS utilizes luciferase-fusion antigens as baits to capture antigen-specific antibodies [30]. Specifically, the target antigens are cloned in-frame with a luciferase reporter gene and expressed in mammalian cells. Crude cell extracts containing the luciferase-tagged antigens are mixed with test serum samples in the presence of protein A Sepharose beads. If the test serum samples contain antibodies specific to the luciferase-tagged antigen, the antigen will be immobilized on the beads. The amount of antigen-specific antibody present in the test serum will be quantified by adding a luciferase substrate, followed by measuring light production (Figure 1). The LIPS has been utilized to measure antibody responses in autoimmune and infectious diseases [31]. In the present study, we demonstrated that the LIPS can be utilized to simultaneously measure swine antibody responses to multiple proteins of PRRSV.

## 2. Materials and Methods

### 2.1. Cell Lines and Reagents

Human embryonic kidney (HEK) 293T cells (ATCC^®^ CRL-3216™) were maintained in Dulbecco’s modified Eagle’s medium high-glucose (Life Technologies, Carlsbad, CA, USA) supplemented with 10% heat-inactivated fetal bovine serum (FBS; Sigma, St. Louis, MO, USA) and penicillin (100 units/mL) and streptomycin (100 µg/mL) (Sigma, St. Louis, MO, USA) (herein designated cDMEM). A mouse monoclonal antibody specific to Nluc was purchased from R&D Systems (Minneapolis, MN, USA). Donkey anti-Mouse IgG (H+L) antibody, Alexa Fluor 488 conjugated, was purchased from Invitrogen (Eugene, OR, USA). DAPI (4′,6-diamidino-2-phenylindole) was purchased from Sigma (St. Louis, MO, USA).

### 2.2. Serum Samples

**Figure 2:**
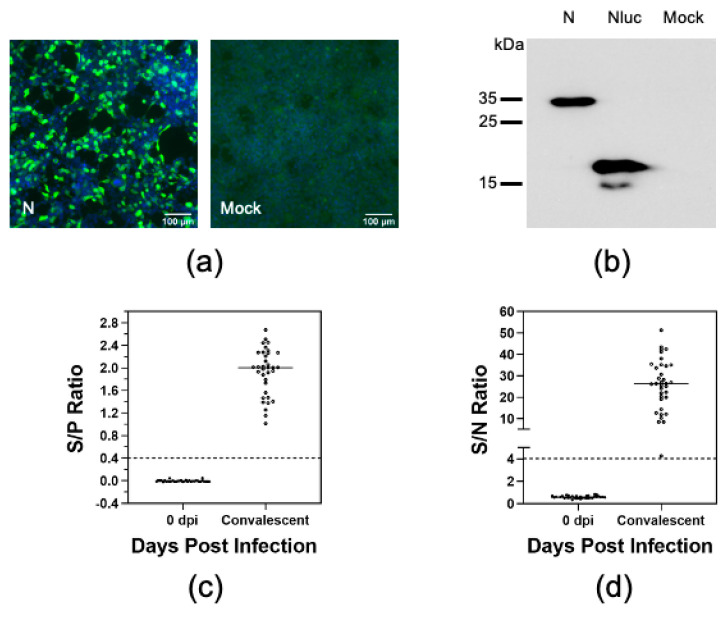
Optimization of the LIPS with N protein. (**a**) HEK-293T cells were transfected with plasmids encoding Nluc-tagged N protein (N) or Nluc only. At 60 h after transfection, cells were fixed and subjected to indirect immunofluorescence assay (IFA) using a monoclonal antibody specific to Nluc protein. The cell nuclear was stained with DAPI (blue). (**b**) HEK-293T cells were transfected as described previously. At 60 h post-transfection, cells were harvested and lysed in RIPA buffer and subjected to immunoblotting analysis using a monoclonal antibody specific to Nluc protein. (**c**) Serum samples collected from 35 pigs experimentally infected with the PRRSV-01 or FL12 and their derivative mutants at 0 dpi and 42 dpi. Serological status of the serum samples as determined by the IDEXX ELISA. Data are expressed as the sample to positive (S/P) ratios. The horizontal dotted line at S/P = 0.4 indicates the cutoff of this assay. (**d**) Immunoreactivity of the serum samples were measured against Nluc-tagged N protein as described in Materials and Method. Fetal bovine serum (FBS) was used as negative control. Data are expressed as sample to negative (S/N) ratios. The dotted line at S/N = 4.02 indicates the cutoff of the assay.

**Figure 3:**
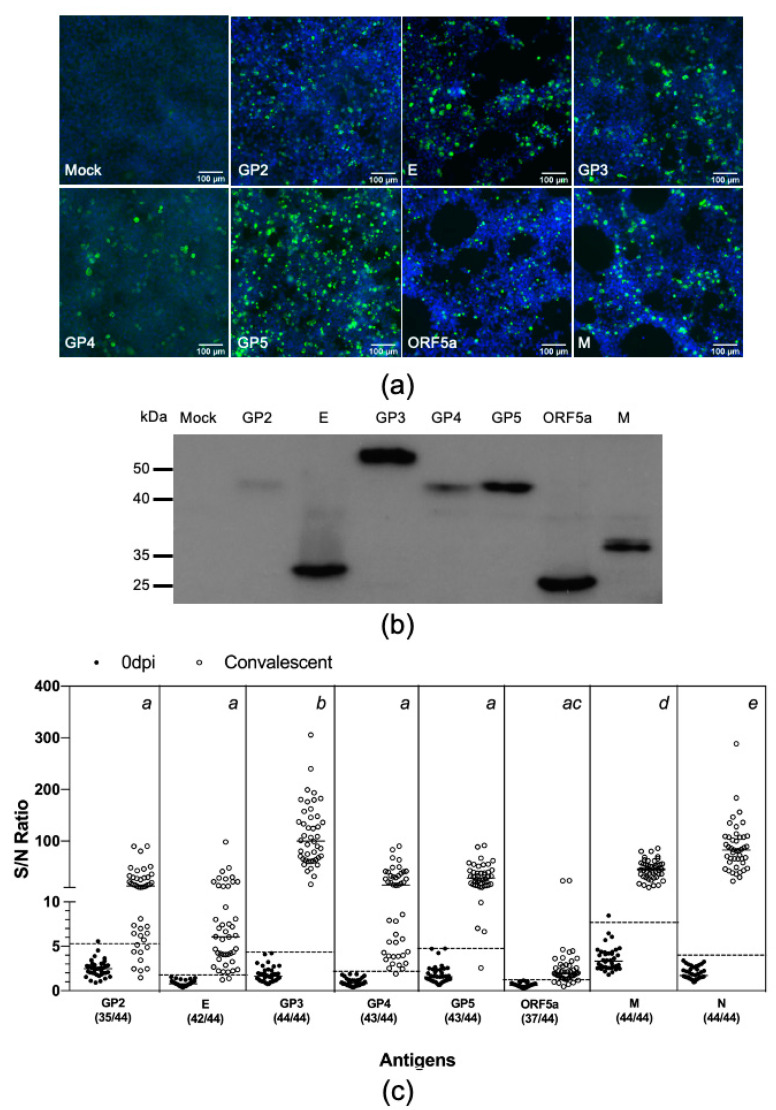
Swine antibody reactivities against PRRSV structural proteins. (**a**,**b**) Evaluation of protein expression. HEK-293T cells were transfected with plasmids encoding Nluc-tagged antigens. At 60 h post-transfection, cells were fixed and subjected to IFA and immunoblotting analysis as described in the Figure 2 legend. (**c**) Thirty-eight serum sample collected before infection (0 dpi) and 44 samples collected at between 42 and 63 dpi from 44 pigs experimentally infected with the PRRSV strain FL12. The antibody reactivities of these serum samples were simultaneous measured against eight PRRSV structural proteins as described in Materials and Method. Fetal bovine serum (FBS) was used as negative control. Data are expressed as sample to negative (S/N) ratios. The dotted lines indicate the cutoffs of the respective assays. The numbers within parenthesis below the antigen names indicate the proportion of convalescent serum samples tested positive for that respective antigens. One-way analysis of variance (ANOVA) was used to evaluate the difference among the mean S/N ratios of the convalescent antisera, followed by Tukey’s multiple comparison test. The superscript letters at the top of the graph denote the statistical comparison of the S/N ratios among the eight antigens. Means sharing the same superscript are not significantly different from each other (*p* > 0.05).

Serum samples from pigs experimentally infected with PRRSV used in this study were collected from previous studies conducted in our laboratory and have been stored at −20 °C [23,28,32,33,34]. In all cases, four-week-old, PRRSV negative pigs were infected intramuscularly with a PRRSV strain at the dose of 10^5.0^ TCID50. For the establishment of the LIPS (Figure 2), we used a set of samples collected at 0 dpi and 42 dpi from 35 pigs including 12 pigs experimentally infected with FL12 and its derived mutants [32] and 23 pigs that were experimentally infected with the PRRSV-01 and its derived mutants [23]. The PRRSV-01 and FL12 derived mutants carry different mutations at the N-linked glycosylation sites in their glycoproteins. For evaluation of antibody response against eight structural proteins (Figure 3) we used 38 samples collected at 0 dpi and 44 samples collected from pigs experimentally infected with the wild-type FL12 between 42 and 63 dpi. For studying the kinetics of antibody responses to GP3 and N protein, we used a set of samples collected from 32 pigs experimentally infected with the wild-type FL12 at 0, 7, 14, and 21 dpi. Field serum samples with known PRRSV serostatus including 84 seronegative and 84 seropositive samples were kindly provided by Dr. Baum at the Iowa State University Veterinary Diagnostic Laboratory.

### 2.3. Plasmid Construction and Protein Expression

The nanoluc luciferase (Nluc) gene sequence (GenBank accession no. JQ437370.1) was modified to incorporate three restriction enzyme sites NheI, EcoRI, and EcoRV to its 5′ end and NotI to its 3′ end. The gene fragment was synthesized by Synbio Technologies (Monmouth, NJ, USA) and cloned into the pCI-neo vector (Promega, Madison, WI, USA) under NheI and NotI. The resulting plasmid, designated pCI-Nluc, was used as the backbone for construction of plasmid expressing Nluc-tagged antigens. Each individual structural gene of the PRRSV strain FL12 was PCR amplified from the pFL12 cDNA clone [35], using a pair of primers listed in Table 1. The resulting PCR products were cloned in-frame to the 5′ end of the Nluc gene in the pCI-Nluc under the restriction enzyme sites NheI and EcoRI. The resulting plasmids were sequenced to verify the authenticity of the inserted genes.

To generate Nluc-tagged antigens, HEK-293T cells were seeded in in 148 cm^2^ tissue culture dishes at the density of 10 million cells in 30 mL of cDMEM. One day later, the cells were transfected with 45 μg of plasmid, using polyethylenimine (PEI) as the transfectant as described previously [36]. At 60 h after transfection, cells were harvested and lysed with 10 mL RIPA lysis and extraction buffer (ThermoFisher Scientific, Rockford, IL, USA), supplemented with 1X protease inhibitor (Pierce Protease Inhibitor Tablet, EDTA-Free, ThermoFisher Scientific, Rockford, IL, USA). The cell lysates were centrifuged at 2000 × g for 10 min and supernatant was passed through a 0.45 μm filter to remove insoluble cell debris. The cell extracts were stored in small aliquots in a −70 °C freezer for future use.

### 2.4. Immunofluorescence Microscopy

HEK-293T cells were seeded onto a 13-mm-diameter glass coverslip that was placed at the bottom of a 12-well plate. The cells were seeded at the density of 1 million cell per well in 1 mL of cDMEM. One day later, the cells were transfected with 1.0 μg plasmid. At 60 h post-transfection, cells were washed with PBS and fixed with a mixture of methanol and acetone (1:1 *v*/*v*) for 15 min at room temperature (RT). The cells were then incubated with an antibody specific to Nluc diluted 1:500 in PBS for 1 h at RT. After three washes with PBS, the coverslips were incubated with Alexa Fluor 488-labeled donkey anti- mouse IgG diluted 1:500 in PBS for 1 h at RT. The coverslips were washed three times with PBS, followed by incubation with DAPI diluted in PBS for 10 min at RT. After three washes with PBS, the coverslips were mounted onto a glass slide in aqueous mounting medium (Sigma, St Louis, MO, USA). Fluorescence images were taken by using a Nikon Eclipse Ti2 which was operated by Nikon NIS Elements (ver 5.02). All images were taken at 20 × using the GFP and DAPI cubes. Images for each filter cube were taken separately and then overlaid to make the final images.

### 2.5. SDS-PAGE and Immunoblotting

HEK-293T cells were cultured in six-well plates at the density of 2 million cells per well in 2 mL of cDMEM. One day latter, the cells were transfected with 2.5 μg plasmid. At 60 h post-transfection, cells were harvested, lysed in RIPA buffer and clarified by centrifugation at 17,000× *g* at 4 °C for 10 min. The cell extracts were mixed with 2 × Laemmli sample buffer and boiled for 5 min. The proteins were resolved by sodium dodecyl sulfate-polyacrylamide gel electrophoresis (SDS-PAGE) and transferred to polyvinylidene difluoride (PVDF) membrane. After 1 h incubation with a blocking buffer (PBS- 0.05% tween-20, 5% skim milk) for 1h at RT, the membrane was incubated with an anti-Nluc antibody diluted 1:1000 in blocking buffer. After five washes in PBS-T20, the membrane was incubated with a goat anti-mouse horseradish peroxidase (HRP)-conjugated secondary antibody diluted 1:2000 in blocking buffer for 1h at RT. After five washes in PBS-T20, protein bands were detected by using an enhanced chemiluminescence (ECL) detection substrate (Pierce Biotechnology, Rockford, IL, USA).

### 2.6. Determination of the Serological Status of the Test Serum Samples

The serological status of swine serum samples was determined by using the IDEXX PRRS X3 ELISA kit (Westbrook, ME, USA), following the manufacturer’s manual.

### 2.7. Luciferase-Immunoprecipitation System

The LIPS was done as previously described [37]. Briefly, test serum samples were diluted 1:40 in buffer A (50 mM Tris, pH 7.5, 100 mM NaCl, 5 mM MgCl2, 1% Triton X-100) and incubated for 1 h at RT on a rocking platform. Fifty μL of each diluted sera was transferred to a well of a 96-well plate, and mixed with 50 μL of the Nluc-tagged antigen extract containing approximately 5 × 10^5^ relative luminometer units (RLU). The plate was incubated for 1 h at RT on a rocking platform. Ten μL of protein A Sepharose 4B (Invitrogen, Camarillo, CA, USA) pre-washed and diluted in 50 uL of buffer A was added to each well of the plate. After another 1 h incubation, the entire content from the 96-well plate was transferred to a 96-well filter HTS plates (EMD Millipore, Billerica, MA, USA) for washing on a vacuum manifold. Each well was washed eight times with 200 μL of buffer A, followed by two times with 200 μL of PBS. After the last wash, 50 μL of distilled water followed by 50 μL of Nano-Glo^®^ Luciferase substrate (Promega, Madison, WI, USA) was added to each well. Luminescence signal was measured by using a Synergy LX multi-mode reader (BioTek, Winooski, VT, USA). Each serum sample was tested in triplicate. Fetal bovine serum (FBS) was used as negative control. Data were expressed as the ratios between the RLU of test serum sample to the RLU of FBS (S/N ratio).

### 2.8. Statistical Analysis

Cutoffs were calculated as mean plus three standard deviations of the S/N ratios of 0 dpi serum samples. Receiver operating characteristic (ROC) analysis was performed to determine the area under the curve (AUC). One-way analysis of variance (ANOVA) was used to evaluate the difference in the S/N ratios among the eight different structural proteins. Two-way ANOVA was used to compare the difference in kinetics of antibody response against GP3 and N protein. Tukey’s honestly significant difference test was used for multiple comparison. The agreement between the detection of PRRSV antibody by the IDEXX ELISA and the LIPS assays was measured using kappa coefficient. Graphs were done using GraphPad Prism (version 8.4.0; GraphPad Software, San Diego, CA, USA).

## 3. Results

### 3.1. Establishment of a Luciferase Immunoprecipitation System to Detect Antibodies against PRRSV

N protein has been used as the target antigen for serodiagnosis of PRRSV [26]. We therefore chose N protein as a model antigen to establish the LIPS to study swine antibody response to PRRSV infection. To produce Nluc-tagged N protein, ORF7 of the PRRSV strain FL12 was cloned in-frame into the 5′ terminus of the Nluc gene in the pCI-Nluc vector (Figure 1a). The resulting plasmid was transfected into HEK-293T cells and protein expression was confirmed by IFA and immunoblotting, using an antibody specific to Nluc (Figure 2a,b).

To establish the assay, we used a set of antisera collected from pigs before (0 dpi) and at 42 days after they were experimentally infected with different PRRSV strains and their derived mutants [23,32]. The commercial IDEXX ELISA was used as a reference test to determine the serological status of these antisera. As expected, all antisera collected at 0 dpi tested negative while all antisera collected at 42 dpi tested positive by the IDEXX ELISA (Figure 2c). The LIPS results show that 42 dpi antisera reacted strongly to the Nluc-tagged N protein, with the S/N ratio of 26.1 ± 11.5 (Figure 2d). On the other hand, the 0 dpi antisera did not react significantly with the Nluc-tagged N protein (S/N 0.6 ± 0.1). A cutoff value was calculated based on the S/N ratio of 0 dpi antisera. Based on this cutoff, all convalescent sera tested positive while all 0 dpi sera test negative by LIPS (Table 2). The LIPS results were highly comparable to the IDEXX ELISA results, with a kappa value of 1.00.

### 3.2. Swine Antibody Response to PRRSV Structural Proteins

We applied the LIPS to comparatively evaluate swine antibody response to all eight structural proteins of PRRSV. The coding sequences for the remaining seven structural proteins—namely GP2, E, GP3, GP4, GP5, ORF5a, and M—were PCR amplified from the FL12 genome and cloned in-frame into the 5′ terminus of the Nluc gene in the pCI-Nluc vector (Figure 1a). The resulting plasmids were individually transfected into HEK-293T cells and protein expression was confirmed by IFA and western blotting, using an antibody specific to Nluc (Figure 3a,b). We noted that the expression levels of the structural proteins were not equal (Figure 3b). Therefore, the protein concentration of the cell lysates was adjusted to ensure that they contained approximately the same relative light units.

Next, we simultaneously measured antibody reactivities against these eight structural proteins using a new set of antisera collected from 44 pigs experimentally infected with the PRRSV strain FL12 between 42 and 63 dpi. Of the eight structural proteins, GP3 had the highest S/N ratio (111.7 ± 59.6), followed by N (87.9 ± 47.2) and M (44.9 ± 18.6) (Figure 3c). The S/N ratios for the GP2 (21.2 ± 22.32), E (11.9 ± 17.1), GP4 (22.6 ±22.8), and GP5 (31.9 ± 20.7) were not statistically different from each other. The S/N ratio for ORF5a-protein (3.1 ± 4.5) was the lowest. Cutoff values were separately calculated for each of these eight proteins. All convalescent antisera tested positive for GP3, M, and N. The percentage of convalescent antisera tested positive for GP2, E, GP4, GP5, and ORF5a-protein were 79.6%, 95.5%, 97.7%, 97.7%, and 84.1% respectively (Figure 3c and Table 3).

### 3.3. Comparison of Kinetics of Antibody Response against GP3 and N Protein

**Figure 4:**
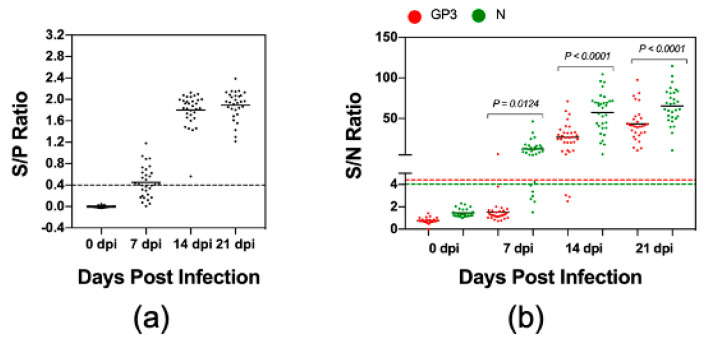
Kinetics of antibody responses to GP3 and N protein. Serum samples were collected from 32 pigs experimentally infected with the PRRSV strain FL12 at 0, 7, 14, and 21 dpi. (**a**) The samples were tested with the IDEXX ELISA. Data are expressed as the sample to positive (S/P) ratios. The horizontal dotted line at S/P = 0.4 indicates the cutoff of this assay. (**b**) The samples were tested by the LIPS with GP3 and N protein as described in Materials and Method. Fetal bovine serum (FBS) was used as negative control. Data are expressed as sample to negative (S/N) ratios. The dotted lines indicate the cutoffs of the respective assays.

Since the convalescent antisera reacted strongest against GP3, we wanted to further evaluate the kinetics of antibody response to this protein. For this experiment, we used a set of serum samples collected from 32 pigs during the first three weeks after they were experimentally infected with PRRSV strain FL12. For comparative purposes, we also analyzed the kinetics of antibody responses to N protein. Again, the IDEXX ELISA was used as a reference test to determine the serological status of these serum samples. Seventeen (53.1%) samples collected at 7 dpi and all antisera collected at 14 and 21 dpi tested positive by the IDEXX ELISA (Figure 4a). For the LIPS with N protein (LIPS-N), 26 (81.3%) samples collected at 7 dpi and 32 (100%) samples collected at 14 and 21 dpi tested positive (Figure 4b). The detection rate for the LIPS with GP3 (LIPS-GP3) was 1 (3.1%) sample at 7 dpi, 29 (90.6%) samples at 14 dpi and 32 (100%) samples at 21 dpi (Figure 4b). The results indicate that pigs experimentally infected with the PRRSV strain FL12 developed antibodies against N earlier than against GP3. In addition to the earlier appearance, the magnitude of antibody responses against N protein was significantly greater than against GP3 (Figure 4b). Interestingly, we noted that the percentage of 7 dpi antisera tested positive by LIPS-N protein was higher than tested with the IDEXX ELISA (81.3% vs. 53.1%).

### 3.4. Measurement of Antibody Reactivities against GP3 and N with Clinical Serum Samples

**Figure 5:**
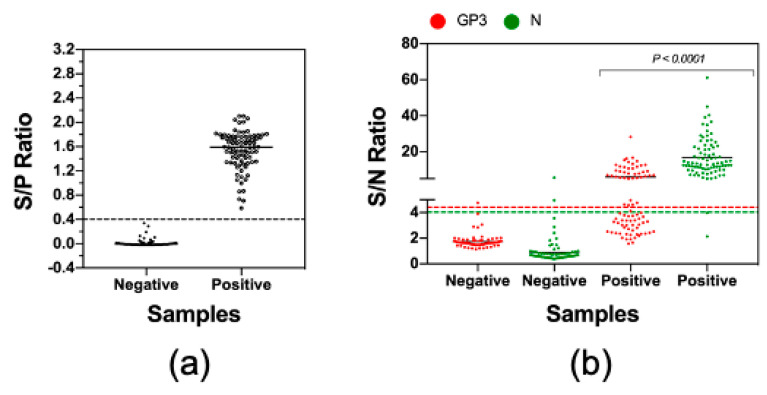
Measurement of antibody reactivities against GP3 and N with clinical serum samples. A set of 84 seronegative and 84 seropositive clinical serum samples were obtained from the Iowa State University Veterinary Diagnostic Laboratory. (**a**) The serological status of these samples was verified by using the IDEXX ELISA. Data are expressed as the sample to positive (S/P) ratios. The horizontal dotted line at S/P = 0.4 indicates the cutoff of this assay. (**b**) The samples were tested by LIPS with GP3 and N protein as described in Materials and Method. Fetal bovine serum (FBS) was used as negative control. Data are expressed as sample to negative (S/N) ratios. The dotted lines indicate the cutoffs of the respective assays.

Finally, we sought to apply the LIPS to examine antibody reactivities against GP3 and N protein in clinical swine serum samples. We were able to collect a set of 168 serum samples which included 84 PRRSV-seropositive and 84 PRRSV-seronegative, as determined by the IDEXX ELISA (Figure 5a). Of the 84 ELISA-negative samples, 82 (97.6%) samples tested negative by LIPS-GP3 and 83 (98.8%) samples tested negative by LIPS-N. Thus, the LIPS assays were highly specific when tested with clinical serum samples. Of the 84 ELISA-positive samples, 40 (47.6%) samples were positive by LIPS-GP3 while 82 (97.6%) samples were positive by LIPS-N protein (Figure 5b). The LIPS-N results strongly agreed with the IDEXX ELISA, with a kappa value of 0.952 while the LIPS-GP3 had moderate agreement, with a kappa value of 0.464 (Table 4).

## 4. Discussion

The LIPS has been widely used for profiling humoral immune responses in autoimmune and infectious diseases owing to its several advantages as compared to other serological assays such as ELISA or protein microarray [30,31]. Briefly, target antigens used for the LIPS are produced in mammalian cells, allowing them to undergo necessary post-translational modifications and proper folding and exposure of the antigenic epitopes. In addition, the LIPS can be performed with crude cell extracts; thus, significantly reducing the time and effort required to produce the target antigens. Renilla luciferase (Rluc) has been widely used as the reporter gene for the LIPS [30,31]. In this study, we chose to use nanoluc luciferase (Nluc) instead of Rluc because Nluc is smaller, brighter, and displays lower background activity than Rluc [38].

We first used PRRSV N protein as the model antigen to validate the LIPS because its immunogenicity has been well characterized [29,39,40,41]. We demonstrated that the LIPS-N provides similar results as compared to the IDEXX ELISA, both with sera obtained from experimentally infected animals and clinical sera obtained from the field. Interestingly, the LIPS-N detected a higher percentage of positive samples than the IDEXX ELISA when it was used to test with serum samples collected at 7 dpi (Figure 4). This is unlikely due to the false positive results because all of the seronegative samples (e.g., serum samples collected from experimental pigs before they were infected with a PRRSV strain and ELISA-negative serum samples collected from the field) tested negative by LIPS-N, demonstrating the high specificity of this assay. Recently, a modified form of the LIPS, namely luciferase-linked antibody capture assay (LACA), was developed for detection of PRRSV-specific antibodies [42]. It was reported in this later study that the LACA was able to detect antibody specific to N protein as early as 3 dpi while the IDEXX ELISA was not able to detect PRRSV-specific antibodies until 7 dpi. Collectively, the available data seem to suggest that the liquid phase immunoprecipitation assay based on luciferase reporter (either LIPS or LACA) might be more sensitive than the IDEXX ELISA for detection of antibody response early after infection.

Once the LIPS was validated, we applied it to simultaneously measure antibody against all viral structural proteins derived from the PRRSV strain FL12. Nsp2 is found to be a component of viral particle and one of the determinants of viral tropism [43,44]. However, we did not include this protein in this study as its immunogenicity has been thoroughly described in multiple studies [29,45]. To avoid the possible influence of mutations of the glycosylation sites on the immunogenicity of the viral glycoproteins, we only used serum samples that were collected from pigs experimentally infected with the wild-type PRRSV strain FL12. Thus, the antibody response against each structural protein measured in this experiment should be considered a homologous antibody response. Equivalent amounts of each of the eight Nluc-tagged antigens were used to measure immunoreactivity. Therefore, the amount of light produced should be proportional to the amount of soluble Nluc-tagged antigens captured by the antibody-bound beads which allows us to directly compare the immunoreactivities of convalescent antisera directed against these eight structural proteins. The results show that GP3, M, and N display the highest degree of immunogenicity. GP2, E, GP4, and GP5 have intermediate levels of immunogenicity while ORF5a protein has the lowest level of immunogenicity.

Although GP5 is a major viral envelop glycoprotein, we observed in this study that the intensity of antibody reactivity against GP5 is not significantly different from the antibody reactivities against the minor structural proteins GP2, E, and GP4. In an effort to identify potential linear B cell epitopes, de Lima et al. screened the reactivity of convalescent sera collected from FL12-infected pigs against a library of overlapping peptides encompassing the viral structural proteins [28]. The authors reported that the frequency and magnitude of antibody reactivity against GP2, GP4, and GP5 peptides were similar. These three proteins carried only one or two linear-B cell epitopes which were recognized by only a fraction (e.g., between 30% and 60%, *n* = 15) of the tested convalescent sera. On the other hand, GP3 and M protein carried multiple linear epitopes that were recognized by 100% convalescent sera [28]. Thus, the results obtained in this current study corroborate the results previous reported by de Lima et al. [28].

ORF5a-protein is a newly discovered viral protein. It is the smallest among the eight structural proteins and is expressed in significantly low levels in PRRSV infected cells [15]. Its immunogenicity has not been well characterized. It was reported previously that antibodies specific to ORF5a-protein slowly appeared in pigs infected and remained at lower levels as compared to antibodies against N protein [15]. In the present study, we observed that immunoreactivity against ORF5a-protein was the lowest among the eight structural proteins (Figure 3c). We suggest that the low immunogenicity of ORF5a-protein might be due to its small molecular weight in combination with its low expression levels.

The finding that GP3 also has a high degree of immunogenicity as compared to M and N protein is interesting. The relative abundance of GP3 on the viral virion is significantly lower than GP5, M, and N [46]. However, it was reported that a portion of GP3 is secreted into culture medium in a soluble membrane-free form [46,47]. It is believed that both virion-associated and soluble-forms of GP3 can elicit immune responses in the infected pigs, which might explain its high immunogenicity [47]. The LIPS-GP3 gave 100% diagnostic sensitivity and specificity when it was used to test samples collected from experimentally infected pigs (Figure 3c). However, the sensitivity of this test significantly reduced when it was used to test samples collect from the field (Figure 5b). Kinetics analysis of antibodies revealed that pigs experimentally infected with the PRRSV strain FL12 developed antibodies against N protein earlier than antibodies against GP3 (Figure 4b). It is possible that clinical serum samples used in this study were collected at early time points after the pigs were infected with PRRSV, when antibodies against GP3 had not yet been generated. In addition, GP3 is more genetically diverse than N protein [48] which might further explain the lower sensitivity of the LIPS-GP3 when it was used to test with field samples.

## 5. Conclusions

In summary, here we report the application of LIPS to simultaneously measure antibody responses against the structural proteins of PRRSV. The results of this study indicate that the levels of immunogenicity are highest with GP3, M, and N; intermediate with GP2, E, GP4, and GP5; and lowest with ORF5a-protein. This study expands our knowledge on the humoral immune response against PRRSV infection.

## Figures and Tables

**Table 1:** Primers used in this study.

Primers	Nucleotide Sequence (5′-3′)
ORF2F	ATATGCTAGCGCCACCATGAAATGGGGTCCATGCAAAGCC
ORF2R	ATATGAATTCCCGTGAGTTCGAAGGAAAAATTGC
ORF2bF	ATATGCTAGCGCCACCATGGGGTCCATGCAAAGCCTC
ORF2bR	ATATGAATTCCATAGCGTCAAGTTGTAAATC
ORF3F	ATATGCTAGCGCCACCATGGCTAATAGCTGTGCACTCC
ORF3R	ATATGAATTCTCGCCGCGCGGCACTGAGAGC
ORF4F	ATATGCTAGCGCCACCATGGCTGCGCCCCTTCTTTTCC
ORF4R	ATATGAATTCAATTGCCAGTAAGATGGC
ORF5F	ATATGCTAGCGCCACCATGTTGGGGAGATGCTTGACCG
ORF5R	ATATGAATTCAAGACGACCCCATTGTTCCG
ORF5aF	ATATGCTAGCGCCACCATGTTCAAGTATGTTGGGGAG
ORF5aR	ATATGAATTCCATAGCGTCAAGTTGTAAATC
ORF6F	ATATGCTAGCGCCACCATGGGGTCGTCTTTAGACGAC
ORF6R	ATATGAATTCTTTGGCATATTTGACAAGG
ORF7F	ATATGCTAGCGCCACCATGCCAAATAACAACGGCAAG ATATGAATTCCGCTGATGATGGCGCTGTG
ORF7R	ATATGAATTCCCGTGAGTTCGAAGGAAAAATTGC

Restriction enzyme sites incorporated in the primers for cloning purpose are underlined. F: forward. R: reverse.

**Table 2:** ROC and kappa coefficient data for LIPS with N protein (LIPS-N).

Characteristics	Value
Optimized cutoff (S/N)	4.02
Diagnostic sensitivity (%)	100.0
95% confidence interval	87.68–100.0
Diagnostic specific (%)	100.0
95% confidence interval	87.68–100.0
AUC	1.0
95% confidence interval	1.0–1.0
Kappa coefficient ^1^	1.0
95% confidence interval	1.0–1.0

^1^ as compared to the IDEXX ELISA.

**Table 3:** ROC and kappa coefficient data for LIPS with eight structural proteins.

Characteristics	Value for Each Structural Protein							
	GP2	E	GP3	GP4	GP5	ORF5a	M	*N*
Optimized cutoff (S/N)	5.32	1.78	4.41	2.24	4.85	1.30	7.741	4.02
Diagnostic sensitivity (%)	79.55	95.45	100.0	97.73	97.73	84.09	100.0	100.0
95% confidence interval	64.25–89.67	83.30–99.21	89.99–100.0	86.49–99.88	86.49–99.88	69.33–92.84	89.99–100.0	89.99–100.0
Diagnostic specific (%)	97.37	100.0	100.0	100.0	100.0	100.0	97.37	100.0
95% confidence interval	84.57–99.86	88.57–100.0	88.57–100.0	88.57–100.0	88.57–100.0	88.57–100.0	84.57–99.86	88.57–100.0
AUC	0.9372	0.9952	1.0	1.0	0.9970	0.9695	1.0	1.0
95% confidence interval	0.8804–0.9940	0.9868–1.0	1.0–1.0	1.0–1.0	0.9903–1.0	0.9338–1.0	1.0–1.0	1.0–1.0
Kappa coefficient	0.758	0.951	1.0	0.976	0.976	0.830	0.975	1.0 ^1^
95% confidence interval	0.620–0.896	0.884–1.0	1.0–1.0	0.928–1.0	0.928–1.0	0.712–0.949	0.928–1.0	1.0–1.0

^1^ as compared to the IDEXX ELISA.

**Table 4:** ROC and kappa analysis for LIPS with field serum samples.

Characteristics	Value for GP3	Value for *N*
Optimized cutoff (S/N)	4.41	4.02
Diagnostic sensitivity (%)	47.62	97.62
95% confidence interval	36.72–58.74	90.86–99.59
Diagnostic specific (%)	98.81	97.62
95% confidence interval	92.63–99.93	90.86–99.59
AUC	0.9636	0.9987
95% confidence interval	0.9365–0.9906	0.9966–1.000
Kappa coefficient	0.464	0.952 ^1^
95% confidence interval	0.349–0.579	0.906–0.998

^1^ as compared to the IDEXX ELISA.
